# Understanding Immune Cells in Tertiary Lymphoid Organ Development: It Is All Starting to Come Together

**DOI:** 10.3389/fimmu.2016.00401

**Published:** 2016-10-03

**Authors:** Gareth W. Jones, David G. Hill, Simon A. Jones

**Affiliations:** ^1^Division of Infection and Immunity, Systems Immunity URI, The School of Medicine, Cardiff University, Cardiff, UK

**Keywords:** tertiary lymphoid organs, ectopic lymphoid structures, lymphoid neogenesis, autoimmunity, infection, rheumatoid arthritis, cancer

## Abstract

Tertiary lymphoid organs (TLOs) are frequently observed in tissues affected by non-resolving inflammation as a result of infection, autoimmunity, cancer, and allograft rejection. These highly ordered structures resemble the cellular composition of lymphoid follicles typically associated with the spleen and lymph node compartments. Although TLOs within tissues show varying degrees of organization, they frequently display evidence of segregated T and B cell zones, follicular dendritic cell networks, a supporting stromal reticulum, and high endothelial venules. In this respect, they mimic the activities of germinal centers and contribute to the local control of adaptive immune responses. Studies in various disease settings have described how these structures contribute to either beneficial or deleterious outcomes. While the development and architectural organization of TLOs within inflamed tissues requires homeostatic chemokines, lymphoid and inflammatory cytokines, and adhesion molecules, our understanding of the cells responsible for triggering these events is still evolving. Over the past 10–15 years, novel immune cell subsets have been discovered that have more recently been implicated in the control of TLO development and function. In this review, we will discuss the contribution of these cell types and consider the potential to develop new therapeutic strategies that target TLOs.

## Introduction

Adaptive immune responses are traditionally viewed as reactions that occur in secondary lymphoid organs (SLOs). These include encapsulated SLOs, such as the spleen and lymph nodes, and mucosal-associated lymphoid tissues, such as Peyer’s patches, nasal-associated lymphoid tissue, bronchus-associated lymphoid tissue (BALT), and tonsils (1). SLOs develop in pre-determined locations throughout the body to monitor self and non-self antigens as they drain from peripheral tissues. Owing to their highly organized cellular architecture, SLOs provide an optimal environment for cellular communication and the generation of antigen-specific effector cells against foreign antigens. In addition, mucosal-associated lymphoid tissues act as tissue barometers responsible for the maintenance of immune homeostasis and orchestrators of anti-microbial host immunity against invading pathogens. They, therefore, reinforce immunological tolerance within mucosal compartments and support tissue integrity through the maintenance of commensal microbiota (2, 3). However, it is increasingly evident that antigen-specific responses may also be generated at sites separate to those SLOs. These responses are typically observed in tissues affected by non-resolving inflammation as a result of infection, cancer, autoimmunity, chronic allograft rejection, and environmental irritants, where the local inflammatory environment promotes the organization of lymphoid aggregates that drive adaptive immune reactions (4). These lymphoid organ-like structures are referred to as tertiary lymphoid organs [TLOs; also called ectopic lymphoid-like structures (ELSs); and ectopic lymphoid follicles (ELFs)].

Unlike SLOs that develop during embryogenesis, TLOs are not encapsulated and do not form in pre-determined locations based on developmental signals (1). Rather, TLOs are inducible in response to inflammatory stimuli and, therefore, have the potential to develop in any tissue where persistent inflammation features. Nevertheless studies in human disease, particularly cancer metastases (5), and research using transgenic mice (6) suggest that some tissues are more permissive to TLO development than others. Furthermore, only a fraction of patients with any particular disease develop TLOs in inflamed tissues. This suggests that the local inflammatory microenvironment, including signals provided by stromal tissue cells and resident cells, must provide specific cues conducive to lymphoid neogenesis for TLO development to occur. Importantly, TLOs can influence disease progression, where their effects can either be beneficial or damaging. For example, in certain cancers and infections, TLOs can promote antigen-specific responses that promote anti-tumor and anti-pathogen immunity (5). However, in autoimmune diseases, such as rheumatoid arthritis, TLOs have been shown to support local autoantibody responses (e.g., rheumatoid factor, ACPA/anti-CCP) linked with disease exacerbation and also influence the clinical response to mainstream biologics (e.g., anti-TNF) (4, 7–9). The above highlight key questions that need addressing: *What are the stromal and immune cell signals that drive TLO development? What determines why some patients develop TLOs during chronic inflammation and others not? What are the most suitable biologics for the treatment of TLO-associated autoimmune diseases? Do TLOs hold promise for establishing anti-tumour immunity to improve cancer therapy? Do signatures of TLO development and activity constitute biomarkers capable of patient stratification that aid clinical decision-making?*

While TLOs borrow developmental cues from secondary lymphoid organogenesis, there are also distinct immune cells, stromal cells, and effector cytokines implicated in TLO development (1, 4). This suggests that during TLO development, immune cells, and their effector molecules can substitute for the traditional players involved in lymphoid organ development. Here, we review recent discoveries relating to the immune cells involved in TLO development, their functions that influence disease progression, and the potential of TLOs as therapeutic targets.

## Secondary Lymphoid Organogenesis as a Model for TLO Development

Secondary lymphoid organs display a highly organized cellular architecture, including segregated T cell zones and B cell follicles comprising active germinal centers (GCs); follicular dendritic cell (fDC) networks; PNAd^+^ high endothelial venules (HEVs) that allow naïve and central memory T and B cell homing; and stromal reticular networks. While TLOs display many of these features, in human diseases they can often present as less ordered structures ranging from simple T and B cell aggregates through to highly organized and segregated structures featuring HEVs and active GCs. This heterogeneity likely reflects the stage at which tissue biopsies are taken and may represent developing TLOs that have not fully matured. Similarly, TLOs can often be “transient” and regress upon successful antigen clearance or resolution of inflammation. Therefore, regression of TLOs may also contribute to the heterogeneity seen in tissues biopsied from human diseases. While TLOs exhibit more heterogeneity than SLOs, much of our understanding of TLO development stems from studies of secondary lymphoid organogenesis [comprehensively reviewed elsewhere (1, 10)].

**Figure 1:**
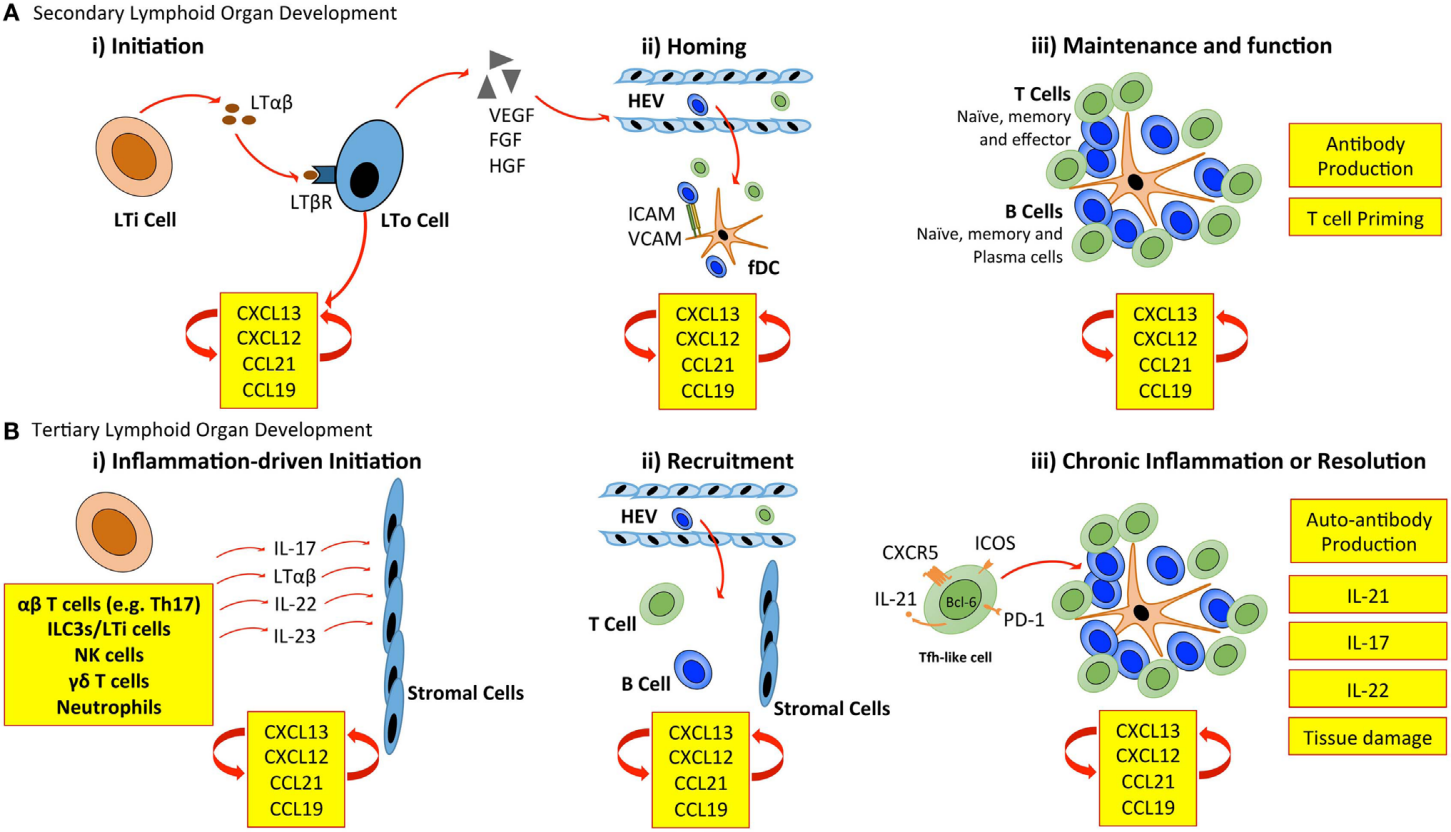
**Cellular control of lymphoid organ development**. **(A)** LTi cells accumulate at sites of SLO development and secrete lymphotoxin (LT) αβ, which engages the LTβ receptor (LTβR) expressed on stromal LTo cells. LTo cells respond by releasing homeostatic chemokines (CXCL13, CXCL12, CCL21, and CCL19), further recruiting and spatially organizing hematopoietic cells into lymphoid organs. Release of vascular endothelial growth factor (VEGF), fibroblast growth factor (FGF), and hepatocyte growth factor (HGF) by stromal LTo cells promotes the development of high endothelial venules (HEVs). Expression of ICAM and VCAM on fDCs and resident stromal cells further supports leukocyte recruitment. Stromal LTo cells are also capable of differentiating into cell lineages (e.g., follicular DCs) that support SLO development. SLOs are sites for T cell activation and differentiation (e.g., into Th1, Th2, Th17, and Tfh cells). Tfh cells promote the development and maintenance of germinal centers and interact with B cells to generate high-affinity antibodies. **(B)** In TLO development, immune cells can substitute for conventional cells involved in lymphoid organogenesis. The accumulation of TLO-initiating immune cells at sites of inflammation, and their interaction with tissue resident and stromal cells initiates release of homeostatic chemokines (CXCL13, CXCL12, CCL21, and CCL19) that are involved in the recruitment and spatial organization of cells into TLOs. The plasticity of T cells may contribute to TLO development through the acquisition of Tfh-like effector characteristics that promote B cell activities and antibody generation. As in SLOs, fDCs may support TLO development and maintenance through chemokine production and provide a cellular network for B cell migration. LTi, lymphoid tissue inducer; LTo, lymphoid tissue organizer; ICAM, intracellular adhesion molecule; VCAM, vascular cell adhesion molecule; fDC, follicular dendritic cell.

Secondary lymphoid organ development is initiated when CD3^−^ CD4^+^ CD45^+^ lymphoid tissue inducer (LTi) cells of hematopoietic origin interact with mesenchymal-derived lymphoid tissue organizer (LTo) cells (Figure 1). LTi cells express the chemokine receptor CXCR5 and IL-7R (CD127), which results in their accumulation at sites of lymph node development in response to the local production of CXCL13 and IL-7. Recruited LTi cells express lymphotoxin (LT)α_1_β_2_, which stimulates stromal LTo cells to produce the homeostatic chemokines CXCL13, CCL19, and CCL21 initiating the recruitment of hematopoietic cells. The retention of cells is further supported by the expression of adhesion molecules, including intracellular adhesion molecule 1 (ICAM-1) and vascular cell adhesion molecule 1 (VCAM-1) by LTo cells (11). The secretion of growth factors, such as vascular endothelial growth factor-C (VEGF-C), fibroblast growth factor-2 (FGF2), and hepatocyte growth factor (HGF) also promotes lymphangiogenesis (the formation of lymphatic vessels from pre-existing lymphatic vessels) and HEV development (1, 12). Finally, LTo cells differentiate into fDCs, and fibroblastic and marginal reticular cells, which form stromal cell networks that provide a structural scaffold that supports cellular migration (13–15). Once initiated, the expression of homeostatic chemokines (CXCL13, CCL19, CCL21, and CXCL12) by LTo cells perpetuates the recruitment of further LTi cells and lymphocytes. This provides a sustained source of LTo cell stimulation through the LTβ receptor (LTβR), thus ensuring the maintenance of lymphoid organ development.

The mechanisms of TLO development share many similarities with those of lymph node development (Figure 1). Perhaps the most prominent example is the establishment of a chemokine-directed positive feedback loop that orchestrates lymphocyte recruitment and organization (4, 5). However, TLOs can form in the absence of LTi cells. For example, mice deficient in the nuclear hormone receptor retinoic acid-related orphan receptor-γt (RORγt) and the transcriptional repressor Id2 still retain the capacity to develop TLOs at inflammatory sites, despite lacking LTi cells (16–19). This highlights one of the most striking differences between TLO and SLO development. While both rely on homeostatic chemokines (e.g., CXCL13, CCL19, CCL21, and CXCL12) and lymphoneogenic cytokines (e.g., LTαβ) for their development, the initiation of TLO development relies on an inducible inflammatory trigger, while SLOs are developmentally pre-programed. In this regard, immune cells may substitute for LTi cells and act as primary orchestrators of tertiary lymphoneogenesis.

## T Helper 17 Type Responses and Plasticity Drive TLO Development in Chronic Inflammation

**Figure 2:**
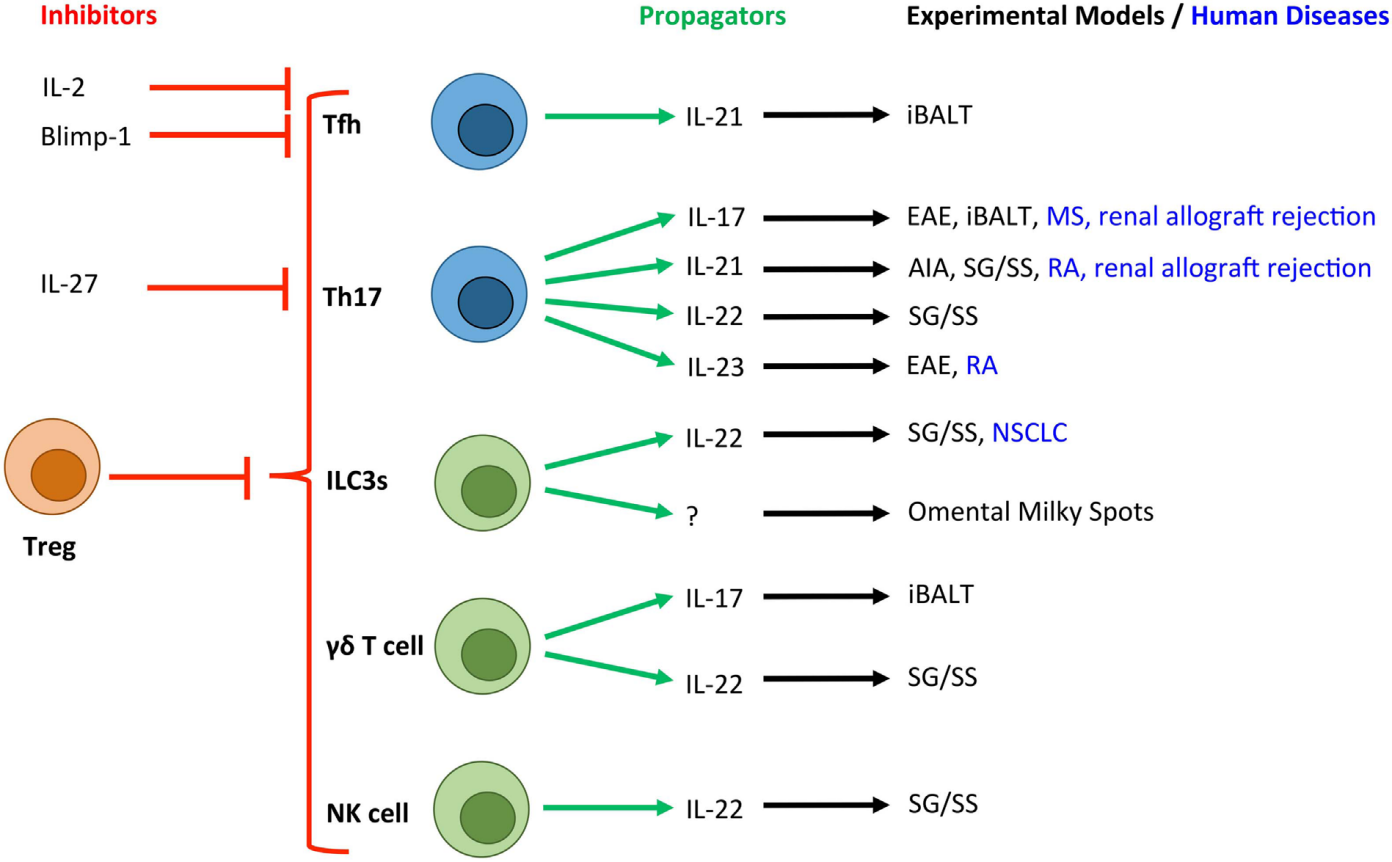
**Novel T cell and innate lymphocyte-derived cytokines associated with TLOs in experimental and human diseases**. Due to the crucial role that T cells (blue) and innate lymphocyte populations (green) play in the development of TLOs, a number of their secretory cytokines have been associated with tertiary lymphoneogenesis. Here, we show the cytokines and transcription factors associated with positive (green arrows) and negative (red arrows) control of TLOs. T cell-derived IL-2 and activation of Blimp-1 result in suppression of Bcl-6 expression and inhibition of Tfh differentiation. IL-27 is produced by activated antigen-presenting cells and potently inhibits the development of Th17 cells, which have been linked with TLO development. Experimental and human diseases where these cells have been linked with TLO development are shown. Tfh, T follicular helper cell; Th17, T helper 17 cell; ILC3s, group 3 innate lymphoid cells; NK cell, Natural Killer cell; Treg, regulatory T cell; EAE, experimental autoimmune encephalomyelitis; AIA, antigen-induced arthritis; iBALT, inducible bronchus-associated lymphoid tissue; RA, rheumatoid arthritis; MS, multiple sclerosis; NSCLC, non-small cell lung carcinoma; SG/SS, salivary gland infection displaying Sjögren’s syndrome-like characteristics.

T helper cells and their effector cytokines, particularly IL-17-secreting CD4^+^ (Th17) cells have recently emerged as key initiators of TLO development in inflammatory diseases (Figure 2) (20). For example, in a model of lipopolysaccharide (LPS)-driven pulmonary inflammation, neonatal mice developed inducible (i) BALT associated with heightened CXCL13 and CCL19 expression (19). Despite the presence of CD3^−^CD4^+^ LTi cells, iBALT development was dependent on IL-17 production by CD4^+^ T cells. Interestingly, antibody blockade revealed that IL-17 was important for initiation of iBALT development, but was dispensable for the maintenance of established lymphoid clusters. The development of iBALT has previously been shown in response to viral challenge (21, 22), bacterial infection (23), cigarette smoke (24), and protein nanoparticles (25). Notably, in response to infection with a replication-deficient poxvirus modified vaccinia virus Ankara, iBALT development was independent of both IL-17A and another Th17 effector cytokine, IL-17F (26). Therefore, while common mechanisms involving IL-17 may promote pulmonary TLO development in response to various inflammatory stimuli, it is also important to note that iBALT formation can occur independently of IL-17/Th17 involvement.

Recent studies have also highlighted roles for Th17 cells in TLO development in the central nervous system (CNS), inflamed joint tissues, and salivary glands. Peters et al. demonstrated a role for Th17 cells in promoting TLOs in an experimental model of multiple sclerosis (27). Here, adoptive transfer of *in vitro* generated Th17 cells induced TLOs, which was partly IL-17 dependent. Only Th17 cells differentiated in the presence of IL-23, which maintains Th17 effector function (28, 29), were capable of inducing TLOs. Notably, the adoptive transfer of Th1, Th2, and Th9 cells failed to induce this phenotype. Interestingly, the development of TLOs in this model was also partly dependent on the expression of podoplanin (gp38) on transferred Th17 cells. While an appreciation of a role for podoplanin in regulating T cell responses is only now emerging (30), there is significant evidence for a role in regulating tertiary lymphoneogenesis. For example, we recently described IL-27 as a negative regulator of TLO development in experimental inflammatory arthritis (31). Here, synovial TLO development in IL-27R-deficient mice was associated with an increased number of peripheral podoplanin-expressing Th17 cells and the local recruitment of podoplanin-positive T cells to synovial lymphoid aggregates. The Th17 axis and podoplanin have also been linked with TLO development in human diseases, including rheumatoid arthritis, multiple sclerosis, renal allograft rejection, and giant-cell arteritis (31–35). Therefore, consistent with a key role for podoplanin and its ligand CLEC-2 in lymph node development (11, 27, 36), podoplanin expression on T cells may support the recruitment and retention of leukocytes within TLOs.

While IL-17 stands as the “signature” cytokine for Th17 cells, these cells also produce IL-17F, IL-22, and IL-21. Recently, IL-17 and IL-22 have been shown to induce stromal production of homeostatic chemokines resulting in TLO development in mucosal tissues (37, 38). For example, IL-22 promotes TLO development in salivary glands following local adenovirus delivery (37). Here, the major source of IL-22 was αβ^+^ T cells and γδ^+^ T cells, which induced the expression of CXCL13 in podoplanin^+^ stromal cells and CXCL12 in epithelial cells. Therapeutic blockade of IL-22 activity inhibited TLO development and maintenance, thus highlighting IL-22-targeted therapies as a novel approach for the treatment of conditions featuring TLOs and autoantibody-driven disease.

IL-21 plays a central role in Th17 and T follicular helper (Tfh) cell differentiation, the development of naïve B cells into plasma cells or GC B cells, and the generation of high-affinity antibodies (39). Therefore Th17 or Tfh cell-derived IL-21 has potential to play an important role in TLO development and function. Elevated expression of IL-21 has been observed in tissues containing TLOs in experimental and clinical rheumatoid arthritis (31, 32), a model of salivary gland inflammation with Sjögren’s syndrome-like characteristics (40) and in human renal grafts undergoing terminal failure (34). A recent study also described the development of TLOs in the retina during experimental uveitis, where TLOs were associated with heightened expression of Tfh cell markers (41). Interestingly, T helper cell plasticity may contribute to the development of TLOs, where T cells may transiently or fully acquire effector characteristics that support tertiary lymphoneogenesis. For example, Th17 cells that migrate and support the development of IgA-producing GC B cells in Peyer’s patches acquire a Tfh-like phenotype, including the expression of IL-21, Bcl-6, CXCR5, and PD-1 (42). Similarly, Th17 cells that promote TLO development in the CNS during experimental autoimmune encephalomyelitis develop Tfh-like effector characteristics (27). While the development of TLOs in this latter model was independent of IL-21, the contribution of other Tfh effector characteristics cannot be excluded. Therefore, plasticity among T helper cell subsets may allow for the acquisition of Tfh-like effector characteristics (43, 44) that can support GC reactions and the development of TLOs.

## Regulatory T Cells as Suppressors of TLO Development and Function

In chronic inflammatory disorders, TLOs are generally considered perpetuators of adaptive immune responses that contribute to pathology. A recent study describing a protective role for TLOs in atherosclerosis (45) raises an interesting question: *How can TLOs in the majority of chronic inflammatory disorders be damaging, yet in some be protective?*

In aged apolipoprotein E (ApoE)-deficient mice with atherosclerosis, smooth muscle cells beneath intimal plaques take on LTo-like properties and secrete CXCL13 and CCL21 to drive tertiary lymphoneogenesis (46). Engagement of the LTβR on smooth muscle cells played a central role in the induction of lymphoneogenic chemokines and the development of aortic TLOs, which were atheroprotective. These TLOs represent principal sites for the regulation of atherosclerotic T cell responses, including the development and activation of anti-inflammatory regulatory T (Treg) cells (45). In these aged mice, highly activated Treg cells within aortic TLOs may skew the local immune response toward anti-atherogenic outcomes by restricting the activation of effector and central memory T cells. Here, communication between aortic TLOs and vascular smooth muscle cells expressing the LTβR may be important in maintaining the structure, size, and composition of TLOs associated with protection from atherosclerosis (45). Notably, another recent study in ApoE-deficient mice outlines a role for regulatory CD8^+^ T cells in limiting the development of aortic TLOs during atherosclerosis (47). However, this study described a pro-atherogenic role for TLOs in both mouse and human atherosclerosis, where Tfh cells support GC reactions and the local maturation of potentially pathogenic B cells. Therefore, while Treg cells can prevent the development of aortic TLOs, further investigation is needed to determine the precise role played by TLOs during atherogenesis. The relative proportions of pro-inflammatory and Treg cell populations within aortic TLOs may be influenced by factors, such as age, stage of disease, and environmental factors, and may ultimately determine the impact of TLOs on disease progression.

Immunotherapies that inhibit Treg cell development or functionality have the potential of supporting antigen-specific responses against tumor antigens and prevent immune evasion by cancer cells. In this regard, many cancer immunotherapies focus on counteracting the immunosuppressive tumor environment to support tumor-specific T cell responses. The presence of tumor-infiltrating Treg cells is linked with immunosuppression and often correlates with poor patient prognosis (48), while TLOs have been associated with improved patient outcomes in certain cancers (5). This raises an interesting question: *Do therapies targeting Treg activities hold potential for supporting local anti-cancer responses at tumour-associated TLOs?* TLO’s have been described in melanoma, mucosal-associated lymphoid tissue lymphoma, and non-small cell lung carcinoma (NSCLC), as well as breast, colorectal, rectal, ovarian, and germ cell cancers [see Dieu-Nosjean et al. (5) for a comprehensive review of TLOs in cancer]. Studies have reported a correlation between the number of TLOs, T/B cell infiltration into tumors and improved patient survival (49–52). In experimental mouse models and human cancers, there is significant evidence that TLOs are functional. For example, in a mouse model of melanoma, tumor-infiltrating lymphocytes developed TLOs and displayed clonal expansion of T cells that are reactive to tumor antigens on melanoma cells and inhibit tumor growth through the release of IFNγ (53, 54). Similar activities were also observed in LTα-deficient mice that lack peripheral lymph nodes, suggesting that T cell responses are primed locally within the tissue (54). In human NSCLC, TLOs are associated with improved patient survival. Here, enhanced anti-tumor immunity is associated with an increased frequency of follicular B cells and plasma cells that display antibody specificity to tumor-associated antigens (50). Therefore, TLO and HEV development in tumors may allow the recruitment of T and B cells that promote GC reactions and anti-tumor immunity.

Some recent studies support the hypothesis that targeting Treg cells can help establish anti-tumor immune responses locally at tumor-associated TLOs. In a mouse model of lung adenocarcinoma, TLOs that included activated Treg cells were observed in ~90% of tumors, where Treg cells suppressed anti-tumor responses (55). In this study, depletion of Treg cells resulted in enhanced expression of costimulatory molecules on dendritic cells (DCs), T cell proliferation, and anti-tumor responses leading to tumor destruction. The development of HEVs, a common feature of TLOs, is also linked with longer remission, reduced metastasis, and improved patient survival (5, 56, 57). Here, HEVs allow for the infiltration of naïve, central memory and effector Th1 cells that support the local priming of antigen-specific tumor responses (53, 58, 59). In a model of carcinogen-induced fibrosarcoma, Treg cell depletion resulted in the formation of HEVs and reduced tumor growth associated with an increase in T cell infiltration into tumors (60). Similarly improved outcomes were seen in a murine model of pancreatic cancer following TGFβ blockade, a cytokine involved in both Treg cell development and effector function (61).

In cancerous tissues, Treg cell populations suppress the proliferative expansion of CD8^+^ T cells responsible for delivering anti-tumor immunity. For example, studies have revealed that depletion of Treg cells contributes to CD8^+^ T cell proliferation and the development of enhanced anti-tumor responses (55, 60). These findings are highly relevant to human forms of cancer where CD8^+^ T cells, which are often found in tumor-associated TLOs, are linked with improved patient outcomes. Interestingly, NSCLC patients with a high frequency of CD8^+^ T cell infiltrates together with a high density of TLOs are associated with significantly improved survival compared to patients with high cytotoxic T cell infiltration without TLOs (51). Thus, tumor-associated TLOs enhance the prognostic value of tumor-infiltrating CD8^+^ T cells. While such studies demonstrate a correlation between CD8^+^ T cell infiltration, TLO densities, and patient survival, they also raise an interesting question regarding the function of cytotoxic T cells: *Are tumour infiltrating CD8^+^ T cells better equipped to control cancer due to their education within tumour-associated TLOs?* While further research is still needed in this area, a recent study of ovarian cancer showed that CD8^+^ tumor-infiltrating lymphocyte responses were increased in the presence of TLOs containing dense accumulations of plasma cells (62). Here, plasma cells expressed markers of active antigen-specific responses and were associated with heightened expression of cytotoxicity-related genes in tumors. TLOs may, therefore, support robust anti-tumor responses, where cytotoxic T cell activity and antibody-secreting plasma cells cooperate to improve patient survival.

Therapeutic strategies that support the development of tumor-associated TLOs and cytotoxic T cell responses may, therefore, prove beneficial for patient treatment. These may include antagonists of Treg cell development, maintenance, or activity. Blockade of TGFβ has been shown to inhibit Treg cells in experimental cancer and autoimmunity (61, 63). Treg cells can also be selectively depleted by metronomic low-dose cyclophosphamide, which improved tumor-specific T cell responses in cancer patients (64, 65). Further strategies may include antibodies, such as ipilimumab that target the CTLA-4 immune-checkpoint receptor, which has been shown to effectively deplete intratumoural Treg cells (66–68). Novel concepts currently in pre-clinical development may also inform next-generation approaches for inducing TLOs in cancer. For example, engineered adjuvant vector cells have been shown to promote the development of TLOs resulting in enhanced antigen-specific T cell responses in pre-clinical cancer models (69). While the majority of studies relating to Treg cells in TLOs are in the cancer field, these cells have also been implicated in suppressing iBALT development in LPS-challenged mice (70) and TLOs in atherosclerosis (45, 47). Where TLOs offer the potential of disease protection, interventions that block Treg cell activities or target their selective depletion may support the development of TLOs and promote anti-cancer or anti-pathogen responses. Such approaches may represent novel routes to patient treatment. However, such immunotherapeutic strategies must delicately balance establishing strong anti-cancer responses with minimizing the development of autoimmunity. This highlights the need to identify approaches for targeting Treg cells within the tumor microenvironment without compromising their role in maintaining immune tolerance.

## Innate Immune Cells Associated with the Development of TLOs

Innate leukocyte subsets have also been implicated in TLO regulation (Figure 2). These include roles for neutrophils (70) and innate lymphoid cells (ILCs) (71–73). Innate lymphocytes have recently emerged as important effector cells with roles in host defense and chronic inflammatory diseases. These cells have been termed ILCs and include cytotoxic ILCs represented by conventional NK cells and three new ILC groups that parallel T helper cell subsets in their cytokine-producing capacity and transcriptional programs (ILC1, ILC2, and ILC3) (74). Of these, ILC3s (which includes both prenatal and adult CCR6^+^ LTi cells) mirror Th17 cells in their expression of the master transcriptional regulator RORγt; the chemokine receptor CCR6; secretion of IL-17, IL-22, and granulocyte-macrophage colony-stimulating factor (GM-CSF); and responsiveness to IL-23 and aryl hydrocarbon receptor (Ahr) ligands (18, 75–77). It is perhaps these similarities in effector characteristics with Th17 cells that result in ILC3s being involved in TLO development (78). For example, adoptive transfer of adult CD4^+^CD3^−^ LTi cells into newborn *Cxcr5*^−/−^ mice, which phenotypically lack Peyer’s patches and isolated lymphoid follicles (ILFs), promoted the development of intestinal lymphoid tissues (73). Here, IL-7 supported the *de novo* generation, proliferation, and survival of LTi cells. In another study, the same group demonstrated that transgenic overexpression of IL-7 supported the development of LTi cells that formed Peyer’s patches, cecal patches and TLOs that displayed functional T cell-dependent B cell responses and GC reactions (72).

Recently, ILC3s have also been associated with tumor-associated TLOs (71). In NSCLC, natural cytotoxicity receptor (NCR)-expressing ILC3s localized to the edge of TLOs and produced IL-22, TNF, IL-8, and IL-2. These NCR^+^ ILC3s interacted with tumor cells and tumor-associated fibroblasts via their NKp44 receptor to trigger production of LTαβ, which resulted in the activation of endothelial cells and mesenchymal stem cells, including upregulation of ICAM-1 and VCAM-1. Thus, ILC3s correlate with the presence of TLOs in NSCLC and may drive the development of lymphoid structures linked with improved patient survival (49).

Innate lymphoid cells have now been associated with TLO development in a number of experimental models of inflammation or infection. While their presence and generation of pro-lymphoneogenic cytokines at TLOs is undisputed, their precise role in tertiary lymphoneogenesis remains to be fully elucidated. For example, ILCs and NK cells contribute to an early production of IL-22 that supports TLO development in salivary glands (37). However, while ILCs may contribute to TLO development in this context, the predominant source of IL-22 in this model were αβ and γδ T cells. Likewise, LTi cells have been found in inflamed lungs that develop iBALT. However, development of these lymphoid aggregates was not reliant on LTi cell activity (19). Intestinal TLOs also develop in response to microbiota in RORγt-deficient mice that lack LTi cells (17). Thus, despite mounting evidence for the presence of ILC3s at TLOs, further research is required to determine a precise role in the initiation of tertiary lymphoneogenesis, where tissue- and disease-specific factors may affect their input. Nevertheless given their presence at TLOs, ILC3s may contribute to the function of TLOs where crosstalk with LTβR-expressing stromal cells and the stimulation of B cells *via* B cell-activation factor (BAFF) and the ligand of costimulatory receptor CD40 (CD40L) has been shown to support antibody production (79–81).

In a similar fashion to ILC3s, γδ T cells can also share effector characteristics with activated Th17 cells, including the secretion of IL-17A, IL-17F, IL-22, IL-21, and GM-CSF (78). While the precise role of γδ T cells in TLO development is also unclear, there is evidence that they contribute to early tertiary lymphoneogenesis. In mice displaying iBALT in response to *Pseudomonas aeruginosa* infection, IL-17 triggered stromal cell differentiation into podoplanin^+^ follicular cells that express CXCL12 (38). Here, γδ T cells were the main source of IL-17. Similarly, development of iBALT in LPS-challenged neonatal mice is also IL-17 dependent, where both γδ and αβ T cells secrete IL-17 (19). Adoptive transfer experiments revealed that while γδ T cells facilitated the development of iBALT, αβ T cells formed larger areas of lymphoid aggregates. It has, therefore, been proposed that an early innate γδ T cell response initiates the development of iBALT, which is later maintained by infiltrating αβ T cells (19, 82). A similar role for γδ T cells in TLO development may occur in salivary glands, where an early prominent IL-22-producing γδ T cell response is later replaced by αβ T cells (37).

Fat-associated lymphoid clusters (FALCs) are TLO-like structures that develop within adipose tissues, including the mesentery, pericardial fat, and milky spots of the omentum (3). Interestingly, milky spots of the omentum increase in number and size during peritoneal dialysis (83, 84). During treatment, catheter insertion, exposure to peritoneal dialysis solution and particularly peritonitis associated with infection results in the expansion and alteration of the cellular composition of milky spots. This suggests an active role for milky spots in peritoneal immunity. Studies in mice demonstrate that the omentum senses peritoneal antigens and represents a site for generating adaptive T cell (CD4^+^ and CD8^+^) and B cell responses, including antibody class switching and somatic hypermutation (85, 86). In these studies, the development of milky spots was independent of LTi/ILC3s and LTαβ, but required stromal CXCL13 for the recruitment of B-1 cells. Interestingly, TNF-expressing myeloid cells, NKT cells, and IL-4R signaling were required for FALC formation following inflammatory challenge (86). This study and others have also demonstrated that FALCs can contain ILC2 cells. For example, during helminth infection, Lin^−^c-Kit^+^Sca-1^+^ cells produce Th2-type cytokines that support B-1 cell proliferation and drive goblet cell hyperplasia (87). Therefore, FALCs, including milky spots, play a role in regulating local immune responses. However, while they display similarities to TLOs, they are often less organized structures with fewer T cells and fDCs and are highly enriched for B-1 cells (3, 85).

Dendritic cells prime adaptive immune responses via antigen processing and presentation to T cells. While DCs are a common feature of TLOs, relatively little is known regarding their precise role in tertiary lymphoneogenesis. This gap in knowledge was addressed by two investigations. First, CD11c^hi^ DCs were identified as being essential for the long-term maintenance and function of iBALT following influenza virus infection (22). Here, depletion of DCs resulted in a loss of LTβ, CXCL13, CCL21, CCL19, and CXCL12 expression that disrupted the structural integrity and cellular organization of iBALT. This was associated with a reduced number of class-switched plasma cells in the lung and a lowering of antiviral serum IgG titers. In a similar approach, Halle et al. used a replication-deficient modified vaccinia virus Ankara to demonstrate that antigen-loaded DCs migrate into iBALT to support the activation of antigen-specific T cells (21). Thus, TLO-associated DCs are a major source of homeostatic chemokines and lymphoid cytokines that support the long-term maintenance of TLOs and encourage the generation of adaptive immune responses through local T cell priming and control of GC reactions.

## Follicular B Cells Drive Autoantibody-Mediated Diseases at TLOs

**Figure 3:**
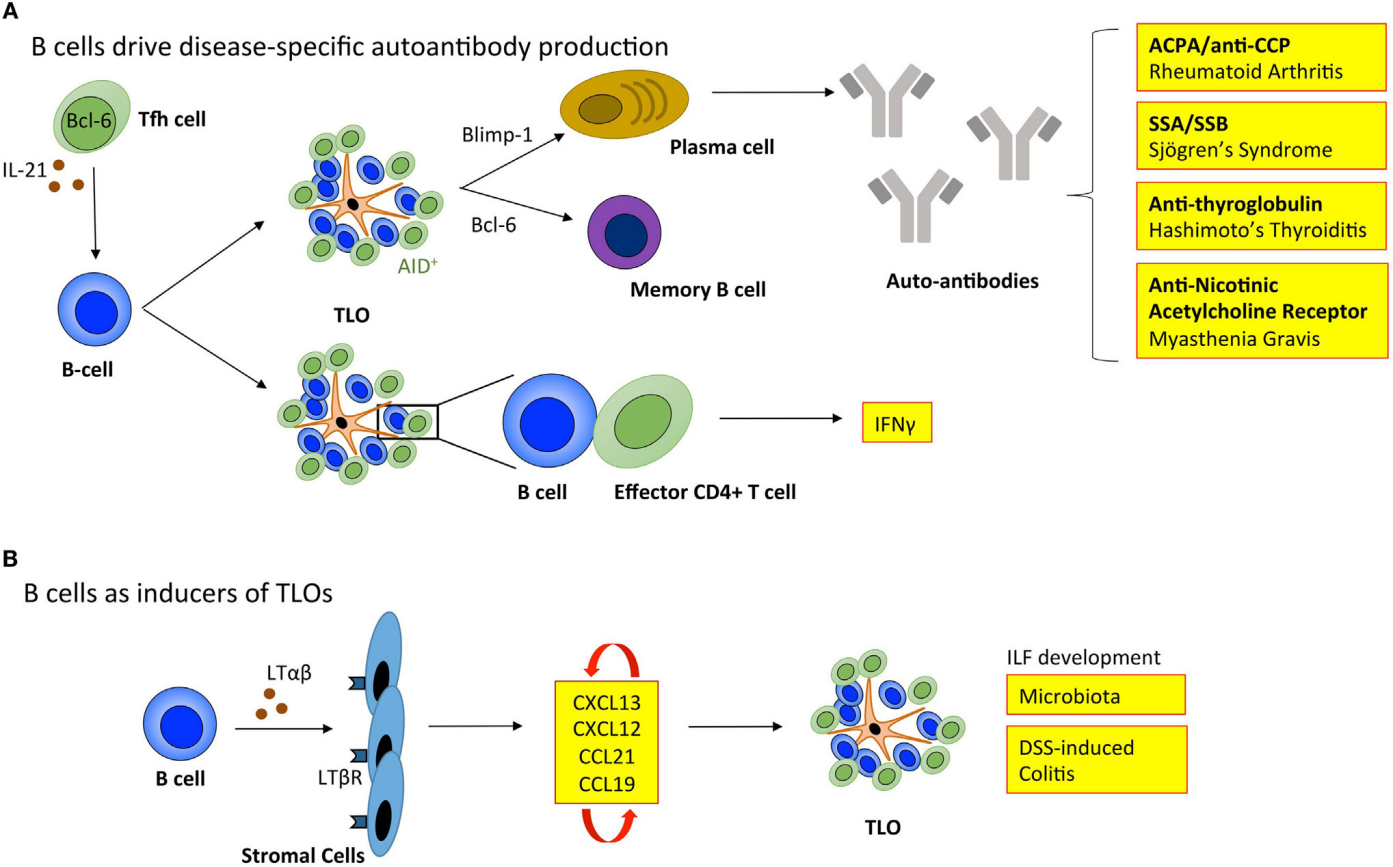
**B cells regulate tertiary lymphoid organ development and function**. **(A)** B cells enable TLOs to recapitulate the function of SLOs. Tfh cells support B cell responses in lymphoid organs. The inverse relationship between Blimp-1 and Bcl-6 governs B cell differentiation into memory B cells or immunoglobulin producing plasma cells. In TLOs, the local generation of disease-specific autoantibodies contributes to disease progression. The expression of activation-induced cytidine deaminase (AID) within TLOs marks active class switching and somatic hypermutation that fine-tunes antibody specificity and effector functions. Also, B cells within TLOs can support T cell effector function, including the secretion of IFNγ. **(B)** B cells can also adopt LTi-like characteristics to promote the development of isolated lymphoid follicles (ILFs) in the small intestine in response to microbiota and dextran sulfate sodium (DSS)-induced colitis. Here, the interaction between B cells and resident stromal cells leads to the production of homeostatic chemokines (CXCL13, CXCL12, CCL21, and CCL19) triggering the recruitment of lymphocytes that initiate lymphoid organ development. Tfh, T follicular helper cell; ACPA/anti-CCP, anti-citrullinated protein antibodies; SSA, Sjögrens syndrome antigen A; SSB, Sjögrens syndrome antigen B; LTαβ, lymphotoxin αβ; LTβR, lymphotoxin β receptor; IFNγ, interferon-γ.

For TLOs to fully recapitulate the function of SLOs they must provide an environment for B cells to undergo affinity maturation and differentiation into memory B cells and antibody-secreting plasma cells (Figure 3). Active GCs express activation-induced cytidine deaminase (AID; also known as AICDA), which promotes somatic hypermutation and class-switch recombination to fine-tune antibody specificity and expand antibody-mediated effector functions (88, 89). In support of TLOs being factories for the development of adaptive immune responses, AID is expressed at TLOs in autoimmunity (7, 90–92), infection (93), and transplant rejection (34). Indeed, analysis of the variable (V)-gene repertoires in TLOs from inflamed tissues reveals a restricted profile of encoded sequences, indicating a clonal expansion of antigen-specific B cells within these lymphoid aggregates (91, 94–96). Furthermore, analysis of Iγ–Cμ and Iα–Cμ circular transcript expression reveals on-going class-switch recombination from IgM to IgG and IgA respectively at TLOs (7, 91, 97). Therefore, significant evidence exists to demonstrate that TLOs can contain functional GCs.

While TLOs may generally be considered protective in infection and cancer, GC activity at TLOs in autoimmunity can result in the local generation of disease-specific autoantibodies that perpetuate disease progression. For example, autoreactive plasma cells release autoantibodies, such as anti-citrullinated protein antibodies (ACPA/anti-CCP) in rheumatoid arthritis (7); antibodies targeting ribonucleoproteins Ro (Sjögren’s syndrome antigen A; SSA) and La (Sjögren’s syndrome antigen B; SSB) in Sjögren’s syndrome (98); anti-thyroglobulin and thyroperoxidase antibodies in Hashimoto’s thyroiditis (99); and nicotinic acetylcholine receptor-specific antibodies in myasthenia gravis (100). The mechanisms that allow autoreactive B cells to accumulate within TLOs (101), when they are efficiently eliminated from GCs in SLOs are currently unclear. In SLOs autoreactive B cells become anergic and are excluded from follicular entry through downregulation of CXCR5, the receptor for CXCL13 (102). Emerging evidence points to a potential role for latent Epstein–Barr virus (EBV) in the development of autoimmunity at TLOs. EBV is a life-long infection, and infected B cells display increased proliferation and survival (103). EBV-infected cells are often observed within TLOs in the inflamed tissues of patients affected by autoimmunity, including rheumatoid arthritis, Sjögren’s syndrome, multiple sclerosis, and myasthenia gravis (92, 104–106). For example, infected B cells display autoreactivity toward citrullinated fibrinogen and ribonucleoprotein Ro, the disease-specific autoantigens for rheumatoid arthritis and Sjögren’s syndrome, respectively (105, 106). Therefore, EBV-infected autoreactive B cells may migrate to target tissues where they differentiate into autoantibody secreting plasma cells and perpetuate autoimmunity.

In addition to their classical role in generating antibody responses, B cells also support local immune cell activation within inflamed tissues. Using an experimental model where synovial biopsies comprising TLOs from rheumatoid arthritis patients are transplanted into severe combined immunodeficient mice (the HuRA-SCID model), Takemura and co-workers demonstrated that depletion of B cells from grafted synovial tissue resulted in a reduction in T cell derived IFNγ production and IL-1β secretion (107). Hence, synovial B cells contribute to T helper cell effector responses in rheumatoid synovitis to influence disease progression.

Typically when addressing the role of B cells in TLOs, the development of antigen-specific antibody responses is the primary consideration. However, B cells have long been known to produce LTαβ, which suggested a potential role in lymphoid neogenesis or the maintenance of lymphoid organs (108). More recently, a role for B cells in promoting the development of ILFs in the small intestine was described (109). Studies using bone marrow chimeric mice demonstrated that LTαβ-producing B cells were required for the development of these TLO-like structures. Similarly, Lochner et al. describe a LTαβ-dependent LTi-like role for B cells in the development of TLOs in dextran sulfate sodium (DSS)-induced colitis (17). This suggests that deploying B cell targeted therapies for the treatment of TLO-associated autoimmune diseases, such as rheumatoid arthritis has the potential to interfere with both early initiation of TLO development and the long-term maintenance of autoantibody responses and local T cell priming. However, in this context, an improved understanding of B cell targeted therapies (e.g., rituximab) is needed, where studies evaluate the peripheral effect on SLOs as well as the local impact on TLOs in inflamed tissues.

## Immune Cell–Stromal Cell Crosstalk is Central to TLO Development and Function

Our understanding of immune cells in the development, function, and maintenance of TLOs has greatly expanded in recent years. While this review is primarily focused on the role of immune cells, it is also important to emphasize the importance of immune cell–stromal cell crosstalk in the development and function of TLOs [comprehensively reviewed elsewhere (110)]. While immune cells adopt LTi-like functions to drive the development of TLOs during inflammation, activated resident stromal cells must phenotypically respond like LTo cells. For example, lung inflammation resulting in the development of iBALT in mice is dependent on immune cell derived IL-17, which triggers CXCL13 and CCL19 expression to recruit and organize lymphocytes (19). While early induction of CXCL13 and CCL19 expression was LTα-independent, once established, homeostatic chemokines and engagement of the LTβR was required for the maintenance of iBALT. Such observations suggest that the dual targeting of both IL-17 and LT signaling may be beneficial for the management of diseases where TLOs are a feature of local pathology. Similar studies using an influenza infection model revealed PNAd^+^ HEVs and stromal cells are the primary source of CXCL13, CCL21, and CCL19 during iBALT development (111). In other infection models that feature TLOs, effector cytokines, such as IL-17 and IL-22, have been shown to drive stromal cell differentiation toward podoplanin-positive CXCL12 and CXCL13 expressing cells (37, 38). Such experimental observations also translate into human disease, where the stromal cell response to inflammation contributes to TLO development. For example, in rheumatoid arthritis patients who display TLOs in inflamed joint tissue, synovial fibroblasts display LTo-like properties including the production of homeostatic chemokines and the induction of BAFF, which supports synovial B cell responses (112–114). Recently, several illustrative examples have further emphasized the significance of immune cell–stromal cell interactions. A highly novel role for TLOs as niches for the maturation of malignant hepatocellular carcinoma progenitor cells was recently described (115). Here, carcinoma progenitor cells exiting TLOs supported tumor growth and outline a detrimental role for TLOs in cancer. Consequently, further work is required to establish the context in which TLOs contribute to the tumor microenvironment. Crosstalk between Th17 cells and stromal cells was also recently shown to be important in an experimental model of multiple sclerosis, where stromal LTβR signaling promoted extracellular matrix deposition, T cell effector cytokine responses, and chemokine production that supported leukocyte retention in the meninges (116). Similarly, in experimental atherosclerosis, vascular smooth muscle cells provided LTo-like function within atherosclerotic aortas to support the development and maintenance of protective TLOs (45). Thus, while immune cells provide a trigger for the development of TLOs in inflamed tissues, the response of stromal cells is equally important in providing an environment conducive to lymphoid neogenesis.

## Concluding Remarks

Studies in recent years have increasingly highlighted potent roles for TLOs in regulating local immune responses in conditions featuring chronic inflammation. Experimental models of disease have provided mechanistic insight and identified novel immune cell subsets involved in TLO regulation, as well as verifying a role for TLOs in pathologic processes. Similarly, studies in human diseases have clearly demonstrated the presence, function, and correlative associations of TLOs with disease severity. However, further research is required to better define the precise role of TLOs in these clinical conditions. Here, it will be important to evaluate the prognostic and diagnostic potential of TLOs in specific diseases, their potential as novel therapeutic targets, as well as to determine how diseases with significant TLO involvement respond to the current arsenal of biologic interventions used in routine clinical practice. In this regard, research into the impact of TLOs in rheumatoid arthritis has provided valuable insight. For example, evidence suggests that rheumatoid arthritis patients with TLOs in inflamed joint tissues display an inferior response to frontline biological therapies that target TNF (8). Therefore, patients with synovial TLOs may be managed better using alternative biological therapies. Given the central role played by TLO B cells in the production of disease-specific autoantibodies, rituximab (an anti-CD20 B cell depleting antibody) may be a better alternative. While further research is needed in this area, two studies oppose this prediction. The first demonstrates that while rituximab treatment results in a reduction of disease-specific autoantibodies (rheumatoid factor and ACPA/anti-CCP) in patient serum, this treatment failed to reduce the local production of these autoantibodies in lymphoid aggregate-containing joint tissue (9). The second study in chronic renal allograft rejection similarly demonstrates that while rituximab treatment depleted peripheral B cells, surprisingly, intragraft B cells in TLOs evaded depletion (117). Thus, B cells residing within the TLO microenvironment may receive signals that allow them to survive rituximab treatment (117). Given the recent emergence of IL-17, and Th17- and Tfh-associated cytokines in the development of TLOs, it will now be interesting to see how targeting these axes fares in the management of TLO-associated diseases. While IL-17-targeted modalities have shown limited clinical efficacy in inflammatory arthritis (118, 119), these clinical trials did not stratify patients for the presence of synovial TLOs. In inflammatory arthritis, sampling of synovial tissue through ultrasound-directed biopsies has become more common in clinical trial design, and have provided new insight into the impact of intervention on TLOs (120). Such approaches may pave the way to identifying optimal strategies for the clinical management of TLO-associated diseases. The success of such approaches may prove important is shaping how clinicians evaluate the diagnosis and treatment of other chronic conditions.

## Author Contributions

GJ, DH, and SJ co-wrote the manuscript and prepared the figures.

## Conflict of Interest Statement

The authors declare that the research was conducted in the absence of any commercial or financial relationships that could be construed as a potential conflict of interest.
